# Nanoscale Mechanical Manipulation of Ultrathin SiN Membranes Enabling Infrared Near‐Field Microscopy of Liquid‐Immersed samples

**DOI:** 10.1002/smll.202402568

**Published:** 2024-08-15

**Authors:** Enrico Baù, Thorsten Gölz, Martin Benoit, Andreas Tittl, Fritz Keilmann

**Affiliations:** ^1^ Chair in Hybrid Nanosystems and Center for NanoScience, Nano‐Institute Munich, Faculty of Physics Ludwig‐Maximilians‐University Königinstr. 10 80539 München Germany; ^2^ Chair of Applied Physics, Molecular physics of life and Center for NanoScience, Faculty of Physics Ludwig‐Maximilians‐University Am Klopferspitz 18 82152 Martinsried Germany

**Keywords:** biospectroscopy, mid‐infrared, PMMA, Scattering scanning near‐field optical microscopy, thin membranes

## Abstract

Scattering scanning near‐field optical microscopy (s‐SNOM) is a powerful technique for mid‐infrared spectroscopy at nanometer length scales. By investigating objects in aqueous environments through ultrathin membranes, s‐SNOM has recently been extended toward label‐free nanoscopy of the dynamics of living cells and nanoparticles, assessing both the optical and the mechanical interactions between the tip, the membrane and the liquid suspension underneath. Here, the study reports that the tapping AFM tip induces a reversible nanometric deformation of the membrane manifested as either an indentation or protrusion. This mechanism depends on the driving force of the tapping cantilever, which is exploited to minimize topographical deformations of the membrane to improve optical measurements. Furthermore, it is shown that the tapping phase delay between driving signal and tip oscillation is a highly sensitive observable to study the mechanics of adhering objects, exhibiting highest contrast at low tapping amplitudes where the membrane remains nearly flat. Mechanical responses are correlated with simultaneously recorded spectroscopy data to reveal the thickness of nanometric water layers between membrane and adhering objects. Besides a general applicability of depth profiling, the technique holds great promise for studying mechano‐active biopolymers and living cells, biomaterials that exhibit complex behaviors when under a mechanical load.

## Graphical abstract

The mechanical and optical interactions between a tapping AFM tip and thin SiN membranes with adhering objects in aqueous environment are studied. Mechanical responses are correlated with simultaneously recorded spectroscopy data to reveal the thickness of nanometric water layers between membrane and sample, a technique which holds great promise for future studies on complex biomaterials such as living cells.

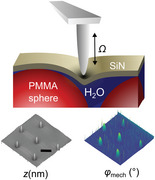

## 1 Introduction

**Figure 1:**
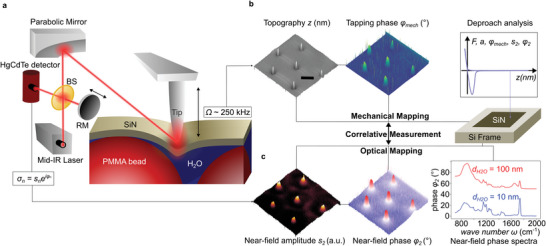
Liquid s‐SNOM setup and associated observables of interest. a) Schematic of SiN‐membrane‐based near‐field probing into liquids using a metal tip operated in tapping mode, able to map the sample's MIR optical response, simultaneously with the mechanical deformation of the membrane caused by the probing tip and objects adhering to the membrane. b) The mechanical channel senses the deflected laser beam (not shown) and returns topography *z* and phase delay *φ_mech_* between cantilever drive and tapping motion. Additionally, tapping‐mode retract curves can be taken to determine both mechanical and optical interactions between the oscillating tip and the thin, pre‐stretched membrane. c) The optical channel senses back‐scattering of the illuminating laser via a Michelson interferometer, which contains a beamsplitter BS and a movable reference mirror RM and returns an optical amplitude and phase (for instrumental details see Experimental Section, for observed results in (b,c) see main text).

Atomic force microscopy (AFM)‐based^[^ ^1^ ^]^ scattering scanning near‐field optical microscopy (s‐SNOM)^[^ ^2^, ^3^ ^]^ using mid‐infrared (MIR) light has become a promising tool for in situ label‐free imaging and spectroscopic studies of biological materials in water at resolutions far below the diffraction limit.^[^ ^4^, ^5^ ^]^ Nanoscale microscopy is achieved by focusing MIR light onto an AFM probing tip (**Figure** **1**a), whereby the tip shaft acts as an optical antenna that creates a self‐focused and strongly confined hot spot below its apex. The achievable resolution of 20 nm^[^ ^6^ ^]^ makes s‐SNOM an excellent tool to image and spectroscopically characterize, for example, subcellular structures of fixed cells.^[^ ^7^ ^]^

Importantly, s‐SNOM operation using a broadband light source^[^ ^8^ ^]^ allows to record nanoscale complex‐valued Fourier‐transform infrared spectra (nano‐FTIR) of a wide range of samples, including liquids,^[^ ^4^, ^5^, ^9^ ^]^ protein aggregates,^[^ ^10^, ^11^ ^]^ and dry^[^ ^12^ ^]^ as well as in‐liquid biological material.^[^ ^5^, ^13^ ^]^ In general, s‐SNOM can conveniently be used to identify any chemical compounds within a submicrometric spot of interest by taking local nano‐FTIR “fingerprint” spectra. The spectra are obtained in the form of amplitude *s_n_* and phase *φ_n_* spectra acquired simultaneously, free of any background after applying demodulation at a suitably high harmonic (n > 1) of the tip's tapping frequency Ω.^[^ ^14^, ^15^ ^]^ The depth of near‐field sensing into a material scales with the tip radius *r*. It is in the range of 100 nm for tips with *r* = 60 nm,^[^ ^5^, ^16^ ^]^ which allows near‐field probing to reach through, for example, biological membranes into the interior of a cell.

Because s‐SNOM is based on an AFM platform, it acquires simultaneously with the optical responses (Figure 1c) the sample's mechanical responses (Figure 1b), which are the surface topography and the mechanical phase delay *φ_mech_* between the signal driving the tapping and the actual oscillation due to the tip interacting with the sample. The latter is a quantity sensitive toward the energy dissipated by the tip tapping above a sample surface.^[^ ^17^, ^18^ ^]^ Various AFM techniques have been introduced to study several cellular properties, such as cell‐cell adhesion forces,^[^ ^19^, ^20^ ^]^ cytoskeleton‐based cell mechanics,^[^ ^21^ ^]^ density of receptors on a cell^[^ ^22^ ^]^ and binding energies between ligands and receptors.^[^ ^23^ ^]^

For s‐SNOM of samples in water (Figure 1a), the use of a SiN membrane has three important, unique advantages, mainly that i) it is highly transparent to the probing MIR near field (depicted as a transparent red ellipsoid), ii) it protects samples from drying by water evaporation (or from contamination/infection through atmospheric constituents), and iii) it provides a stable, flat and homogenous surface for the scanning tip.^[^ ^5^ ^]^ As demonstrated in our previous study on in situ nano‐imaging of living cells and microparticles adhering in water below a 10‐nm thin SiN membrane, such objects can induce additional, topographical contrasts as well as tapping phase contrasts through nanoscale deformations of the membrane.^[^ ^5^ ^]^ Since our s‐SNOM (see Experimental Section) stores all optical and mechanical observables simultaneously, it serves as a highly correlative nano‐imaging technique.

Here, we assess the mechanics of the flexible, 10 nm thin membrane freely spanned across a solid frame in contact with water by measuring the reversible indentation induced by a tapping AFM tip through tip‐retraction effects on the tapping oscillation with a systematic variation of tapping parameters such as the tapping amplitude. We further investigate the case where submerged, spatially fixed and well‐defined PMMA spheres with 10 µm diameter adhere and support the membrane by recording mechanical and optical images at varying tapping parameters. Despite the SiN membrane fully covering the sample, we find that a wealth of sub‐membrane information can be extracted by considering the deformation of this thin covering sheet.

Furthermore, we show that the SiN membrane deformation is controllable through a simple variation of the tapping parameters. We find that the tapping phase *φ_mech_* is the observable to determine the mechanical properties of a sample adhering to the thin SiN membrane, being a measure for the energy dissipated through the tip‐sample system, and we show that lower tapping amplitudes are preferable to maximize tapping phase contrast while minimizing topographical deformation.

Lastly, we correlate mechanical and optical data to enable depth profiling of the thin water layer that may form between sample and membrane. We find that, apart from a dimple, a bulge can also be created in the membrane by the tapping tip, which can induce artefacts in measured near‐field signals due to the deformation decreasing the water layer's thickness underneath the membrane. In order to counteract this effect, we present a viable protocol to determine the optimal tapping parameters in order to minimize the deformation of the membrane, while simultaneously maintaining a high quality of spectral acquisition and high mechanical phase contrast. In this way, the method allows to determine the ideal tapping amplitude to avoid cross‐talk between optical and mechanical measurements and to measure the near‐field signal of the sample underneath the membrane without artificial signals created through topographical deformations.

This technique could in the future be straightforwardly extended to other, more complex biological materials, such as proteins or lipids, which could be studied in more detail in order to investigate their prompt response to external manipulation, for example, by changing the mechanical pressure exerted by the AFM tip, or by changing the pH value, or the salt concentration of the solution. Our study aims to combine thin‐membrane mechanics and near‐field optics for improving future s‐SNOM investigations of nanoscale objects in aqueous environment.

## 2 Results and Discussion

### 2.1 Mechanical Interactions between s‐SNOM Tip and SiN Membrane

When recording tapping‐mode retract curves, the initial in‐contact tapping amplitude *a*, is the decisive parameter. In most s‐SNOM experiments, it is chosen between 20 and 100 nm. Typical tapping frequencies are 250–350 kHz. The effective force applied to the membrane by the oscillating tip is an average over changing forces encountered during an oscillation cycle. By contrast, when taking contact‐mode force‐distance curves, the tip interacts with the surface for much longer durations, thus allowing short‐range repulsive forces to have greater impact. For a detailed description of the mechanics of the interactions between the tip and the thin pre‐stretched membranes and of the contact models that can be employed to describe them, see Section S1 (Supporting Information).

**Figure 2:**
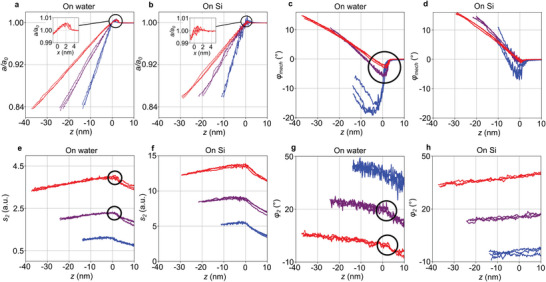
Tapping‐mode retract curves from the 10‐nm SiN membrane either on water or on Si frame. a,b) Tapping amplitude *a/a_0_*, c,d) tapping phase *φ*_mech_, e,f) optical amplitude *s_2_* (at the peak of water absorption, 1650 cm^−1^) and simultaneously recorded g,h) optical phase *φ*_2_ at three different tapping amplitudes *a* = 60 nm (blue), *a* = 120 nm (purple) and *a* = 200 nm (red). The zero on the z‐axis is set to where the amplitude *a* reaches *a_0_*, i.e., its constant value beyond the loss of contact. Anomalies (encircled, inset) suggest a repeatable tip‐induced bulging upward of the membrane.

Tapping‐mode retract curves were taken, either on Si or on water at 90% of the free cantilever's resonance frequency *Ω_0_* and at a set point of 85% of the free cantilever's amplitude *a_0_*. For a given tapping amplitude *a*, the measurements of all four mechanical and optical observables were taken simultaneously versus *Z*‐piezo position *z* (**Figure** **2**).

The curves taken on water (Figure 2a) show an unexpected effect, which is not present or not as pronounced in those taken on the Si frame (Figure 2b): the tapping amplitude *a* rises above *a_0_* for *x* > 0, indicated by a black circle and an inset). Our interpretation is that this retract region is dominated by adhesion forces from the tip that pull the membrane upward until it snaps back. The same effect becomes visible also in the simultaneously recorded tapping phase *φ_mech_* (encircled in Figure 2c), which shows an even more abrupt snap back. This observation adds to our former demonstration with living cells^[^ ^5^ ^]^ that the tapping phase is especially sensitive for mapping local contrasts of adhering objects. In addition, the optical amplitude (Figure 2e) and optical phase (Figure 2g) keep developing continuously with *a* past *x* = 0, until they experience abrupt changes in behavior (black circles), as can be expected for a sudden opening of an air gap between tip and sample. Measurements on the Si frame (Figure 2b,d,f,h) do not exhibit the same trend. In particular, the inset in Figure 2b taken on bulk Si also shows a small, noisy rise of *a/a_0_* beyond 1, likely due to weak attractive forces between the tip and Si surface, but it is much smaller since it can be assumed that the tip does not deform the solid Si frame during a retract curve.

In addition to measurements shown in Figure 2, which were conducted in tapping mode, we recorded retract curves on both Si and SiN membrane in contact mode, the latter with and without water adhering underneath (Figure S2, Supporting Information). The retract curves shown in Figure S2 (Supporting Information) reveal that the membrane deforms almost identically for both the measurements with and without water adhering to the membrane's underside. This allows to determine an effective elastic modulus and spring constant by fitting Kirchhoff plate theory^[^ ^24^ ^]^ (see Section S1 and Equation (S2), Supporting Information) to the curves recorded on the pre‐stretched SiN membrane (Figure S2a, Supporting Information). From these curves, we obtain an effective spring constant of the membrane‐water system of *k_m,eff_* = 6·10^−3^ Nm^−1^ and an effective elastic modulus of the SiN membrane with water adhering underneath of *E_m_,_eff_* = 21 TPa. This value far exceeds that of previous studies on SiN thin films, such as a study conducted by Yuxing et al.,^[^ ^25^ ^]^ where values between 190 and 320 GPa were obtained. We suggest that this discrepancy may be due to not considering the pretension present on the membrane, which can drastically affect the value of the calculated Young's modulus (see Section S2, Supporting Information).

### 2.2 S‐SNOM Mechanical Nano‐Imaging of Submerged Objects

**Figure 3:**
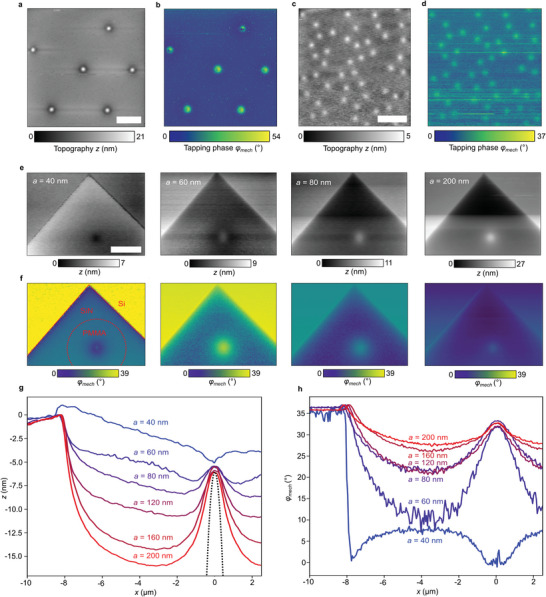
Mechanical maps of hexagonally arranged PMMA microspheres in water. a,c) Topography (*Z*‐piezo position) *z* and b,d) tapping phase *φ_mech_* for spheres of diameter 10 and 2 µm, respectively, adhering below a 10 nm thin SiN membrane. e) Topography (*Z*‐piezo position) and f) tapping phase images of a single 10‐µm PMMA sphere near a Si corner, from left to right at *a* = 40, 60, 80, and 200 nm. All scale bars are 5 µm. For simultaneously recorded optical images see overview images in Figure 1b,c. g) Topography profiles and h) mechanical profiles of *φ_mech_* extracted from e,f demonstrate that the tip is pulling the membrane upward when *a* is chosen <60 nm. The dotted curve in g depicts the shape of a 10 µm PMMA sphere.

**Figure 4:**
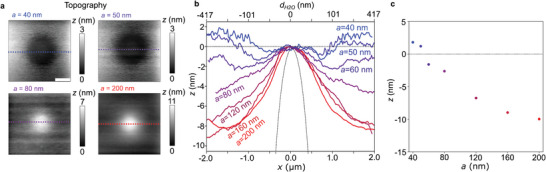
Topography reversal at low tapping amplitudes. a) Topography of a single 10 µm PMMA sphere in water at different values of *a* as indicated, Scale bar 1 µm. b) Profiles extracted from dotted lines versus *x* (lower axis), or respectively, nominal water thickness *d_H2O_* (upper axis), defined by $d_{H2O}=R_{PMMA}\;-\;\sqrt{R_{PMMA}^{2}-\;x^{2}}$, where *R_PMMA_* is the sphere radius. The dotted curve depicts the PMMA sphere. c) Extracted height difference *z* between the sphere center and far away (2 µm), for *a* between 40 and 200 nm.

**Figure 5:**
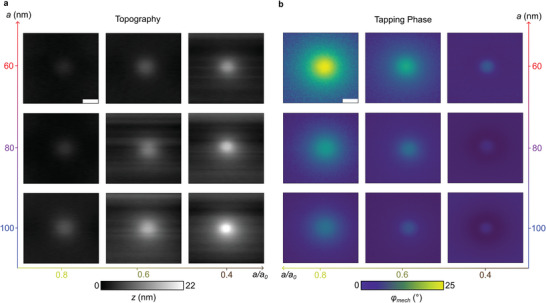
Influences of tapping amplitude and setpoint. a) Measured topography and b) tapping phase map of a 10‐µm PMMA sphere in water adhering underneath a 10‐nm SiN membrane, assembled in a 3 × 3 matrix with varied tapping amplitudes (vertical) and amplitude setpoints (horizontal). Extracted profiles are shown in Figure S5 (Supporting Information).

Our former experiments have revealed that a thin membrane covering water deforms under the mechanical load of an AFM tip,^[^ ^5^ ^]^ an effect demonstrated across the edge of the Si‐frame at a single tapping amplitude *a*. Here, we study how this deformation depends on *a* using an aqueous sample with the membrane supported by PMMA spheres (10 or 2 µm diameter) close to the Si frame's edge (**Figure** **3**). With *a/a_0_* set to 0.8, we acquire a series of AFM images (topography *z* and tapping phase *φ_mech_*) for *a* between 40 and 200 nm (Figure 3a–f). The PMMA spheres are arranged in hexagonal patterns, each creating a bulge in the membrane surface, as can be seen in the topography images. We conclude that the spheres form self‐organized 3D hexagonal crystals fixed to the Si frame and to the supporting pre‐tensioned membrane. The crystals seem strong enough that the tip's tapping forces do not affect their structure. Note that the topography *z* shown in Figures 3, 4, 5 corresponds to the *Z*‐piezo position.

The tapping forces clearly induce a reversible dimple in the membrane at almost any point of probing. The topography in Figure 3a,c appears flat between spheres (of both sizes), from which we deduce that the dimple's depth is constant on deep enough water. However, at the points where the spheres are in contact with the membrane, the lattice structure provides stability and thus reduces the membrane's flexibility. At the center of each sphere, the deformation fully vanishes, which can be concluded from the fact that the topographical height *z* there becomes independent of the tapping amplitude *a*. Altogether, the PMMA lattice supports the pre‐tensioned membrane and enables to extract information on tip‐induced topographical deformations.

The mechanical tapping phase *φ_mech_*, on the other hand, effectively measures the total dissipated energy at each pixel of a sample's surface^[^ ^26^ ^]^ (see Section S3, Supporting Information, for details). Figure 3b,d,f shows that *φ_mech_* increases on stiffer samples, in agreement with our earlier report (Figure 2c in Ref. [5]). Remarkably, decreasing tapping amplitudes results in increasing the *φ_mech_* contrast between an adhering particle and its aqueous surrounding (Figure 3h). This observation clearly shows that low tapping amplitudes enhance the mechanical sensitivity of detecting local changes of energy dissipated by the tip tapping above the SiN membrane. Figure 3g further shows that topographical deformations can be almost fully eliminated just by choosing a suitably small tapping amplitude. The achievement of a structureless topography would, in principle, suppress any topography‐induced crosstalk in optical near‐field images. In addition, the use of small tapping amplitudes is a well‐known advantage of better suppressing optical background‐scattering signals.^[^ ^2^ ^]^

**Figure** **4** shows the tip‐induced membrane deformation profiles in the vicinity of a single 10 µm PMMA sphere in more detail. The membrane evidently exhibits a dimple or a bulge, depending on the value of *a*, which form on water somewhat away from the PMMA sphere. For *a* = 60 nm, the membrane stays approximately flat, while for lower tapping amplitudes, the membrane is being pulled upward. In Figure 4c, we extracted bulge/dimple heights *z* versus.

As mentioned before, the SiN membrane's upper surface may be covered by a thin water film, whose thickness can be expected to increase with the atmosphere's relative humidity, which could, through capillary forces, strongly attract the tip to the surface.^[^ ^27^ ^]^ This mechanism may explain why we are not able to achieve stable tapping with *a* below 20–30 nm, as the tip gets trapped on the surface and is unable to oscillate properly, or even crashes into the membrane. This could also be responsible for the observed increased fluctuations in the measured retract curves at small tapping amplitude (Figure 2b,d, blue for *a* = 60 nm).

The full set of images from which the data in Figure 4b has been extracted is shown in Figure S3 (Supporting Information). Surprisingly, the topographies at *a* = 40 and 50 nm exhibit rings which display a shallow minimum around the sphere's center. These rings may indicate the highly interesting existence of a critical thickness of confined water on the order of tens of nm, below which the attraction of the membrane by the tip is overcompensated through adhesion forces between membrane and PMMA sphere. While the physical origin of this observation is beyond the scope of the present study, we tentatively suggest that i) a rheological contribution could become important with ultrathin water layers, and that ii) the widely observed changes of the water's supramolecular structure next to surfaces may play a role.

The effect of increasing membrane deformation for higher tapping amplitudes almost fully vanishes when the sample is dried out (Figure S4, Supporting Information). Then, the particle forms a permanent bulge of ≈80 nm height, irrespective of the tapping amplitude used for imaging. We attribute this bulge to non‐volatile contaminants of the PMMA suspension which accumulate during the process of drying on the underside of the membrane, thereby permanently deforming the membrane. This also explains the strong local deformations of the membrane visible across the entire topographical scan in Figure S4a (Supporting Information), as opposed to the relatively flat surface seen in prior measurements.

In order to find the tapping parameters yielding maximum phase contrast, we varied both tapping amplitude *a* and amplitude setpoint *a/a_0_* in a 3 × 3 matrix of mechanical images of the same PMMA sphere (**Figure** **5**). It is well known in the AFM literature that decreasing the amplitude setpoint increases the tip's time‐averaged loading force^[^ ^28^ ^]^ and results in detuning the driving frequency *Ω* further down from the free cantilever resonance frequency *Ω_0_* toward a lower frequency. Additionally, as can be seen from quantitative, extracted profiles shown in Figure S5 (Supporting Information), lower amplitude setpoints induce deeper dimples in the membrane away from the sphere, as do higher tapping amplitudes *a*. On the other hand, higher setpoints and lower tapping amplitudes both lead to higher tapping phase contrasts while leaving the membrane almost flat.

### 2.3 Correlating Infrared Spectroscopic and Mechanical Information

**Figure 6:**
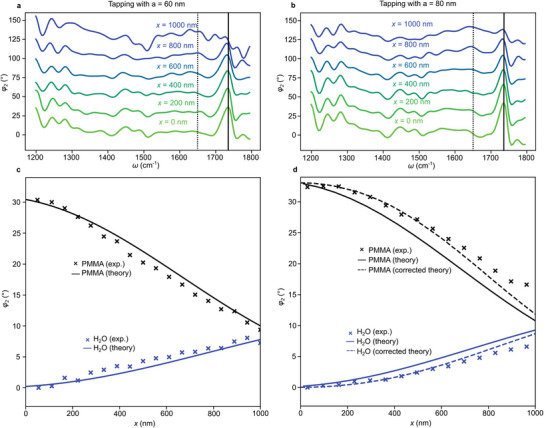
Topography‐corrected subsurface near‐field probing. a,b) *φ*_2_ spectra taken at radial distances x from the starting position (*x* = 0) above a 10‐µm PMMA sphere's center, using *a* = 60 or 80 nm respectively, show key vibrational resonances assigned to carbonyl (at 1735 cm^−1^, solid line) and to the bending vibration of *H_2_O* (at 1650 cm^−1^, dotted line). Curves are offset for clarity by 25° each. c,d) Plots of extracted peak *φ*_2_ signals for PMMA (black crosses) and water (blue crosses) versus radial distance *x*, using *a* = 60 nm or 80 nm respectively, and calculated theory curves (full and dashed curves, see text for details).

We identify and assess a possible cross‐talk mechanism between mechanical and optical responses for membrane‐based s‐SNOM in aqueous environments, where membrane deformation may influence the optical near‐field signal of a sample adhering to the membrane. To investigate this effect, we extend our previous approach of spectroscopically distinguishing stacked sub‐membrane layers^[^ ^5^ ^]^ by recording nano‐FTIR phase spectra *φ*_2_ along a radial line, starting from a 10‐µm PMMA sphere's center, for two different tapping amplitudes *a =* 60 nm and *a =* 80 nm (**Figure** **6**a,b). The choice of these values ensures stable tapping operation, whereas tapping at, for example, *a* = 40 nm often fails because the cantilever gets trapped on the sample. Even though, as shown in Figures 3 and 4, high tapping amplitudes have an inherent disadvantage of yielding lower tapping phase contrasts, we show in the following experiments that the relatively small difference between 60 and 80 nm can have drastic effects on the cross‐talk between mechanical and optical responses.

Quantitative complex spectra on PMMA and on water shown in Figure S6 (Supporting Information) were used to define a characteristic MIR frequency, 1735 and 1650 cm^−1^, for each material respectively. We extracted phase signal heights at both of the characteristic frequencies from each spectrum in Figure 6a,b and plotted the spectral weight of both materials versus radial position *x* shown in Figure 6c,d.

For a theoretical prediction, we first assume a plane membrane touching the sphere at a single point, from which it follows that the nominal water thickness *d_H2O_* increases with x, according to a formula given in the caption of Figure 4. The finite dipole model of s‐SNOM^[^ ^29^, ^30^, ^31^ ^]^ extended to a 3‐layer sample (see Experimental Section) then yields the dependencies shown in Figure 6c,d (full curves, phases at *x* = 0 were multiplied by 1.05 to match the measured values).

Interestingly, the theoretically calculated curves match the measured points better at *a* = 60 nm than at *a* = 80 nm. For an explanation, recall from Figures 3 and 4 that for *a* = 60 nm, the membrane is almost completely flat, while for *a* = 80 nm, a sizable dimple forms outside the sphere center, which means that the thickness of the water space there is smaller than *d_H2O_*. Therefore, the signal of the carbonyl resonance at 1735 cm^−1^ comes out higher than theoretically expected. Since the deformation of the membrane is known from recorded topography images, we can calculate a corrected theoretical prediction by simply replacing *d_H2O_* by *d_H2O_* – *z*, and indeed obtain curves that fit the data much better (dashed in Figure 6d). Clearly, using topographical data to analyze infrared‐spectroscopic measurements of multilayer samples can be of great value and should, in particular, enable accurate depth profiling of processes in aqueous environments.

The smallest feasible value (here *a* = 60 nm) to minimize the topographical deformation of the membrane not only depends on the dimensions of the tip and the viscoelastic properties of the sample and liquid adhering underneath the membrane but also on external factors such as the atmospheric relative humidity. The latter affects the thickness of a water film on the upper surface of the SiN membrane that can strongly influence tip‐sample interactions through capillary forces. Generally, our method of conducting initial test scans at varied tapping conditions should be useful to i) determine optimal tapping parameters in many experimental situations in order to minimize cross‐talk between optics and mechanics, to ii) attain background‐free near‐field signals, and to iii) maximize tapping phase contrast, all of which, in our case, are best fulfilled by choosing *a* = 60 nm.

Correlating infrared‐spectroscopic nano‐imaging with AFM data not only works for rigid PMMA spheres but can certainly be applied to more elastic biomaterials. As an example, we present measurements on aggregated amyloid‐beta protein strands in water in Figure S7 (Supporting Information). Interestingly, the topography is repeatably measured to be almost fully flat over multiple hours. Additionally, the aggregated proteins are clearly visible in the near‐field optical signal simultaneously recorded, yielding repeatable mechanical and optical contrasts and illustrating the potential to use this technique for various kinds of biological and chemical applications. When investigating sub‐membrane samples in future studies, the methods used in this work should prove essential for correctly determining the thickness of water layers between membrane and adhering objects and retrieval of the sample's tomography without significant cross‐talk between optics and mechanics.

## 3 Conclusion

Our measurements of membrane topography and tapping‐phase nanoscale images, supported by modeling, clearly show that the tip‐induced deformation of a thin SiN membrane separating air and water half‐spaces can be controlled by two tapping parameters, amplitude *a* and setpoint *a/a_0_*. Unexpected features seen in tapping‐mode retract curves reveal a novel mechanism of manipulating the membrane, pulling it upward and forming a water‐filled bulge. This mechanism is simultaneously documented in the development of four different mechanical and optical observables. Abrupt changes in these developments when the tip reaches a certain height indicate a sudden appearance of an air gap between tip and membrane due to the collapse of the bulge when the membrane loses contact to the tip and snaps back. Furthermore, our measurements reveal that the tapping phase *φ_mech_* is the most sensitive observable to detect mechanical changes and thus represents the key for unlocking new insights on the mechanical behavior of adhering samples in water, such as migrating living cells.

Furthermore, our AFM imaging of hexagonally arranged PMMA spheres in water provides a guideline to achieve highest tapping phase *φ_mech_* contrast: small tapping amplitudes and high setpoints. A welcome side effect is that small tapping amplitudes and high setpoints minimize tip‐induced deformations of the covering SiN membrane, which is highly desirable to avoid topographical artefacts in simultaneously recorded near‐field optical signals. Accomplishing high tapping phase contrasts for in‐liquid samples would allow for future studies of highly interesting dynamic processes, such as the mechanics of cell adhesion to elastic surfaces or local variations of stiffness on the surface of a living cell when exposed to chemicals that alter its architecture.^[^ ^32^, ^33^, ^34^ ^]^

Infrared near‐field probing through the membrane and a thin water layer into a PMMA sphere adhering to the membrane allows to quantitatively assess spectra as well as the local thickness of the water. Using this technique, future experiments may contribute to our knowledge of structure and dynamics of asymptotically thin water spaces, enabling s‐SNOM to be utilized as a tool for subsurface tomography, as has been done in previous studies with dry samples.^[^ ^14^, ^35^ ^]^ Such experiments can be conducted to retrieve both the dielectric functions and thicknesses of a number of layers through the acquisition of near‐field spectra and fitting the results using the finite dipole model.^[^ ^31^ ^]^ By obtaining both topography and optical amplitude/phase images and ensuring that the tapping parameters are chosen such that the topography stays approximately flat, one can assess a sample's near‐field response unaffected by topographical deformation, avoiding errors in tomographical determinations.

We also observed that nano‐FTIR is able to resolve and characterize single sub‐micrometer protein aggregates in water (Figure S7, Supporting Information), which we assume to be much less rigid than PMMA, showing that the method is applicable for both hard and soft matter systems. Future experiments on biological samples following the protocols established here could thereby prevent tip‐induced damage to the sample, or detachment from the deformed membrane due to the energy dissipated by the tapping tip, which may be challenging for objects much smaller than 100 nm, in the range of 10 nm comparable to the membrane deformations shown in this work. This capability could potentially unlock the key to answer the long‐standing open question in biology, of revealing the mechanism (including dynamics) of prionic infections at the single‐prion level.^[^ ^36^, ^37^ ^]^

Finally, the methods introduced in this work can be directly applied when extending membrane‐based s‐SNOM studies by a microfluidic system which allows exchanging the aqueous medium while keeping the membrane‐adhering nanoscale objects in place. In particular, pH value, salt concentration, gas content and specific biochemical agents could be varied in short time and with greatly sub‐ml expenditure, without disturbing the objects' position, orientation, and nanometer‐scale resolved morphology.

## 4 Experimental Section

### AFM‐Based Scattering Scanning Near‐Field Optical Microscopy

In this work a commercially available s‐SNOM (NeaSNOM from *attocube systems*, Haar, Germany) was used, operated in nano‐FTIR mode. Hereby, the tip (nano‐FTIR tip, from *attocube systems*) was illuminated by a broadband MIR coherent source based on difference‐frequency generation of <100 fs fiber laser pulses (FemtoFiber dichro midIR NEA 31002 from Toptica Photonics). The source emits an 800 cm^−1^ wide spectrum and was centered at 1300 cm^−1^. The optical path of the s‐SNOM features a ZnSe beamsplitter, a paraboloidal focusing mirror, a liquid nitrogen‐cooled HgCdTe‐detector (IRA‐20‐00103 from InfraRed Associates) and a second parabolic mirror for focusing the back‐scattered light onto the detector's active area of 0.0025 mm^2^. The beamsplitter (BS) sends part of the incident beam to a reference mirror (RM) on a moving stage to form an asymmetric Michelson interferometer, where it interferes with the back‐scattered beam. The resulting interferogram yields a complex‐valued scattering coefficient *σ_n_* containing both amplitude *s_n_* and phase *φ_n_* information, demodulated at the nth harmonic of the tip oscillation frequency *Ω* in order to eliminate background‐scattering signals. This allows to either record the total scattered light from the tip (white light imaging), or to acquire local nano‐FTIR spectra by periodically moving the RM.

### Sample Preparation in Liquid Cell

The liquid cell used in this work^[^ ^5^ ^]^ consists of a metal plate with a central hole of 2 mm diameter that was covered by fixing, with double‐sided adhesive tape, a commercial Si chip carrying a freestanding SiN membrane (NX5025Z, Norcada, Edmonton, Canada). The metal holder was 2 cm in length and width and had a height of 0.3 cm, meaning that, with an opening of 0.4 cm, the total volume of liquid that could be contained was around 30–40 µL. In addition, the depth of the holder far exceeds both the size of the microparticles used in this study and the near field's penetration depth and thus could be assumed to be semi‐infinite for the purposes of near‐field measurements. The membrane was 250 × 250 µm^2^ in size and 10 nm thick, with an elasticity given by the manufacturer of *E* = 270 GPa and a pretension of *T_pre_* <250 MPa (www.norcada.com). The membrane was UV‐irradiated for 30–40 min to ensure sufficient hydrophilicity of the SiN surface for the liquid sample to wet the surface of the membrane. Subsequently, ≈20 µL of a 1:10 diluted 10 µm‐diameter PMMA microsphere solution (PMMA‐F‐10.0, microparticles GmbH, Berlin, Germany) was dropcast into the metal holder and incubated for 15 min. The adherence of microparticles and water to the membrane was checked by visual inspection with an optical microscope. In the end, the chip was sealed with a glass cover slide to prevent water from evaporating.

### Computations and Data Processing

All analytical calculations and data processing of experimental results in this work have been computed using Python. To predict the optical response of multilayered materials analytically, the finite dipole model was employed, extended to multi‐layer objects by the transfer matrix method.^[^ ^30^ ^]^ It is an excellent and computationally effective tool that requires only knowledge of the tip geometry and the layer's dielectric function, in the case for SiN,^[^ ^38^ ^]^ water,^[^ ^39^ ^]^ and PMMA.^[^ ^40^ ^]^ For the calculations, the PMMA particles are assumed to have a perfect spherical geometry. The model parameters used are *L* = 300 nm, *g* = 0.6, *r* = 60 nm.

## Conflict of Interest

F. K. is a scientific advisor to attocube systems AG which manufactures the s‐SNOM used in this study. T. G. obtained financial support for his PhD thesis from attocube systems.

## Author Contributions

E.B. prepared samples and performed s‐SNOM and nano‐FTIR experiments. F.K. and T.G. conceived the idea and implemented the SiN liquid cell. E.B., T.G., and F.K. analyzed the data. E.B. conducted model calculations. E.B., M.B., A.T., and F.K. interpreted the results. E.B. and F.K. wrote the paper. All authors contributed to the scientific discussion.

## Supporting information

Supporting Information

## Data Availability

The data that support the findings of this study are available from the corresponding author upon reasonable request.

## References

[1] G. Binnig , C. F. Quate , C. Gerber , Phys. Rev. Lett. 1986, 56, 930. 10033323 10.1103/PhysRevLett.56.930

[2] F. Keilmann , R. Hillenbrand , Philosophical trans. Series A, Mathematical, Phys., Eng. Sci. 2004, 362, 787. 10.1098/rsta.2003.1347 15306494

[3] A. Zayats , D. Richards , Nano‐optics and near‐field optical microscopy, Artech House, Boston, London 2009, p. 361.

[4] Y.‐H. Lu , J. M. Larson , A. Baskin , X. Zhao , P. D. Ashby , D. Prendergast , H. A. Bechtel , R. Kostecki , M. Salmeron , Nano Lett. 2019, 19, 5388. 31306028 10.1021/acs.nanolett.9b01897

[5] K. J. Kaltenecker , T. Gölz , E. Bau , F. Keilmann , Sci. Rep. 2021, 11, 21860. 34750511 10.1038/s41598-021-01425-w PMC8576021

[6] F. Huth , A. Govyadinov , S. Amarie , W. Nuansing , F. Keilmann , R. Hillenbrand , Nano Lett. 2012, 12, 3973. 22703339 10.1021/nl301159v

[7] K. Kanevche , D. J. Burr , D. J. Nürnberg , P. K. Hass , A. Elsaesser , J. Heberle , Commun. Biol. 2021, 4, 1341 34848821 10.1038/s42003-021-02876-7 PMC8633277

[8] S. Amarie , T. Ganz , F. Keilmann , Opt. Express 2009, 17, 21794. 19997423 10.1364/OE.17.021794

[9] Y.‐H. Lu , C. Morales , X. Zhao , M. A. van Spronsen , A. Baskin , D. Prendergast , P. Yang , H. A. Bechtel , E. S. Barnard , D. F. Ogletree , V. Altoe , L. Soriano , A. M. Schwartzberg , M. Salmeron , Nano Lett. 2020, 20, 6364. 32786946 10.1021/acs.nanolett.0c01801

[10] L. M. Meireles , I. D. Barcelos , G. A. Ferrari , P. A. A. A. Neves , R. O. Freitas , R. G. Lacerda , Lab Chip 2019, 19, 3678. 31570906 10.1039/c9lc00686a

[11] I. Amenabar , S. Poly , W. Nuansing , E. H. Hubrich , A. A. Govyadinov , F. Huth , R. Krutokhvostov , L. Zhang , M. Knez , J. Heberle , A. M. Bittner , R. Hillenbrand , Nat. Commun. 2013, 4, 2890. 24301518 10.1038/ncomms3890 PMC3863900

[12] H. Amrania , L. Drummond , R. C. Coombes , S. Shousha , L. Woodley‐Barker , K. Weir , W. Hart , I. Carter , C. C. Phillips , Faraday Discuss. 2016, 187, 539. 27077445 10.1039/c5fd00150a

[13] O. Khatib , J. D. Wood , A. S. McLeod , M. D. Goldflam , M. Wagner , G. L. Damhorst , J. C. Koepke , G. P. Doidge , A. Rangarajan , R. Bashir , E. Pop , J. W. Lyding , M. H. Thiemens , F. Keilmann , D. N. Basov , ACS Nano 2015, 9, 7968. 26223158 10.1021/acsnano.5b01184

[14] L. Mester , A. A. Govyadinov , S. Chen , M. Goikoetxea , R. Hillenbrand , Nat. Commun. 2020, 11, 3359. 32620874 10.1038/s41467-020-17034-6 PMC7335173

[15] L. Mester , A. A. Govyadinov , R. Hillenbrand , Nanophotonics 2022, 11, 377. 10.1515/nanoph-2021-0565 PMC11501567 39633877

[16] P. McArdle , D. J. Lahneman , A. Biswas , F. Keilmann , M. M. Qazilbash , Phys. Rev. Research 2020, 2, 023272.

[17] J. Tamayo , R. García , Langmuir 1996, 12, 4430.

[18] J. Tamayo , R. García , Appl. Phys. Lett. 1998, 73, 2926.

[19] M. Benoit , D. Gabriel , G. Gerisch , H. E. Gaub , Nat. Cell Biol. 2000, 2, 313. 10854320 10.1038/35014000

[20] P.‐H. Puech , K. Poole , D. Knebel , D. J. Muller , Ultramicroscopy 2006, 106, 637. 10.1016/j.ultramic.2005.08.003 16675123

[21] G. Zhou , B. Zhang , G. Tang , X.‐F. Yu , M. Galluzzi , Adv. Phys.: X 2021, 6, 1866668.

[22] H. Kim , H. Arakawa , T. Osada , A. Ikai , Ultramicroscopy 2003, 97, 359. 12801689 10.1016/S0304-3991(03)00061-5

[23] V. T. Moy , E. L. Florin , H. E. Gaub , Science 1994, 266, 257. 7939660 10.1126/science.7939660

[24] A. E. H. Love , Phil. Trans. R. Soc. A 1888, 179, 491.

[25] R. Yuxing , D. C. Lam , Mater. Sci. Eng., A 2007, 467, 1.

[26] G. Bar , R. Brandsch , M. Bruch , L. Delineau , M.‐H. Whangbo , Surf. Sci. 2000, 444, L11.

[27] A. Schirmeisen , B. Anczykowski , H. Fuchs , in Nanotribology and Nanomechanics, Vol. 2 (Ed: B. Bhushan ), Springer, Berlin, Germany 2008, Ch. 6.

[28] J. Legleiter , Nanotechnology 2009, 20, 245703. 19471079 10.1088/0957-4484/20/24/245703

[29] N. Ocelić , Doctoral Thesis, Technische Universität München, 2007. Univ. München e.V.

[30] B. Hauer , A. P. Engelhardt , T. Taubner , Opt. Express 2012, 20, 13173. 22714345 10.1364/OE.20.013173

[31] A. Cvitkovic , N. Ocelic , R. Hillenbrand , Opt. Express 2007, 15, 8550. 19547189 10.1364/oe.15.008550

[32] S. Pérez‐Domínguez , S. G. Kulkarni , C. Rianna , M. Radmacher , Neuroforum 2020, 26, 101.

[33] C.‐A. Lamontagne , C. M. Cuerrier , M. Grandbois , Cell Mol. Physiol. 2007, 456, 61. 10.1007/s00424-007-0414-0 18080817

[34] A. Krüger , A. Bürkle , K. Hauser , A. Mangerich , Nat. Commun. 2020, 11, 2174. 32358582 10.1038/s41467-020-15858-w PMC7195430

[35] A. A. Govyadinov , S. Mastel , F. Golmar , A. Chuvilin , P. S. Carney , R. Hillenbrand , ACS Nano 2014, 7, 6911. 10.1021/nn5016314 24897380

[36] S. B. Prusiner , Proc. Natl. Acad. Sci. USA 1998, 95, 13363. 9811807 10.1073/pnas.95.23.13363 PMC33918

[37] K. M. Pan , M. Baldwin , J. Nguyen , M. Gasset , A. Serban , D. Groth , I. Mehlhorn , Z. Huang , R. J. Fletterick , F. E. Cohen , Proc. Natl. Acad. Sci. USA 1993, 90, 10962. 7902575 10.1073/pnas.90.23.10962 PMC47901

[38] G. Cataldo , J. A. Beall , H.‐M. Cho , B. McAndrew , M. D. Niemack , E. J. Wollack , Infrared dielectric properties of low‐stress silicon nitride, Opt. Lett. 2012, 37, 4200. 10.1364/OL.37.004200 23073410

[39] J.‐J. Max , C. Chapados , J. Chem. Phys. 2009, 131, 184505. 19916610 10.1063/1.3258646

[40] A. Röseler , Infrared Spectroscopic Ellipsometry Akad.‐Verl., Berlin, 1990.

