# Supplementary material for: Nanoscale Mechanical Manipulation of Ultrathin SiN Membranes Enabling Infrared Near‐Field Microscopy of Liquid‐Immersed samples
Source: Small. 2024 Aug 15;20(47):2402568. doi: 10.1002/smll.202402568 (PMC11579970; doi:10.1002/smll.202402568)
Supplement: Supplementary file 1 — Supporting Information [file SMLL-20-2402568-s001.pdf]

NANO · MICRO
small

Supporting Information

for *Small*, DOI 10.1002/smll.202402568

Nanoscale Mechanical Manipulation of Ultrathin SiN Membranes Enabling Infrared Near-Field Microscopy of Liquid-Immersed samples

Enrico Baù, Thorsten Gölz, Martin Benoit, Andreas Tittl and Fritz Keilmann*

Supplementary Information

Nanoscale mechanical manipulation of ultrathin SiN membranes enabling infrared near-field microscopy of liquid-immersed samples

Enrico Baù[1], Thorsten Gölz[1], Martin Benoit[2, 3], Andreas Tittl[1,*], and Fritz Keilmann[1]

1. Chair in Hybrid Nanosystems, Nano-Institute Munich, Faculty of Physics, Ludwig-Maximilians-University, Königinstr. 10, 80539, München, Germany

2. Chair of Applied Physics, Molecular physics of life, Faculty of Physics, Ludwig-Maximilians-University, Am Klopferspitz 18, 82152 Martinsried, Germany

3. Center for NanoScience, Ludwig-Maximilians-University, Amalienstr. 54, D-80799 München, Germany

1. Mechanics of an AFM tip interacting with a pre-stretched thin membrane

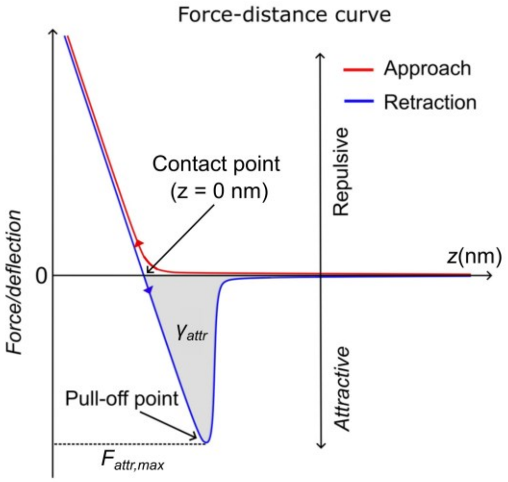

Figure S1. Schematic force-distance curve. During the approach (red) of the AFM tip towards the sample surface, the contact point will be reached, followed by a steady increase of the repulsive force. The retrace curve (blue) shows that, after passing a point where both attractive and repulsive forces balance each other such that the total force extended to tip and cantilever vanishes, the tip enters an attractive regime, until reaching the pull-off point, where contact between tip and sample is lost. The grey area under the curves yields the adhesion energy and the maximum adhesion force is determined at the pull-off point.

To understand the mechanics of the SiN membrane, we analyze the indentation caused by the tapping tip. In general, the motion of an AFM tip interacting with a surface can be described as a driven damped oscillator system, taking into

account attractive and repulsive forces between tip and sample [1]. These forces include long-range Van-der-Waals, electrostatic and short-range repulsive forces and are heavily dependent on the sample and tip material properties.

The equation of motion of a tapping AFM probe can be expressed as:

$$m\frac{d^2z}{dt^2} + \frac{m\Omega_0}{Q}\frac{dz}{dt} + kz = F_0\cos\Omega t + F_{TS} \quad (1)$$

where m denotes the mass of the probe, Ω_0 its resonance frequency and Ω the frequency when in contact with the sample, Q the quality factor, k the spring constant of the oscillating AFM cantilever, F_0 the driving force and F_{TS} the sum of interaction forces between tip and sample. Furthermore, z denotes the position of the Z-piezo.

For both contact-mode and tapping-mode approach and retract curves, the AFM tip tapping from above deforms the sample, indenting it until it reaches its lowest point, as shown in **Fig. S1** (red curve). Subsequently, the AFM tip is retracted (blue curve) until the indentation vanishes at the $F = 0$ nN line. Beyond this point and until the pull-off point is reached, adhesion forces dominate. The greyed-out area in between the $F = 0$ nN line and retraction curve estimates the total work of adhesion. The maximum adhesion force is reached at the pull-off point. Note that this behavior is present both for in-contact and tapping mode indentation curves, although when operating in tapping mode, the bulge caused by a tip tapping (at Ω~300 kHz in our case) on a sample surface results from a time-average of all loading forces during a full oscillation cycle, rather than in case of the contact mode, by a stable tip which relatively slowly approaches or pulls away from the sample.

Calculating exact quantitative–forces involved in AFM measurements require extensive computations [2]. One approach to quantify the mechanics of suspended thin membranes is Kirchhoff plate theory [3]. Previous publications [4, 5, 6, 7, 8] dealing with stretched thin sheets have treated tip-sample interactions using either numerical computations or simplified analytical versions of Kirchhoff plate theory. In these simplified versions of the model, a point load force F acts on a suspended membrane with radius R_m and thickness t_m. If the membrane is sufficiently thin and $t_m/R_m \ll 1$, the model describes the relation between applied force F and indentation δ in the following way [8]:

$$F = \frac{4\pi E}{3(1-\nu^2)}\frac{{t_m}^3}{{R_m}^2}\delta \quad (2)$$

Additionally, to remove the cantilever contribution and retrieve the effective elasticity of the SiN membrane with water adhering underneath, a force-distance curve on a rigid material such as Silicon can be recorded. The angle of the slope fitted to the data points with respect to the y-axis can then be considered to be the contribution of the cantilever bending, thus the curves taken on water can be multiplied by this angle in order to eliminate this contribution.

2. Contact-mode retract curves

We recorded force-distance curves in contact mode on a 10 nm SiN membrane either with liquid underneath or on the rigid Si-frame (**Fig. S2**). 40 individual retract curves were taken during 60s each (velocity of 17 nm/s) and then averaged to obtain the curves shown below, showing *Z*-piezo position *z* vs. force *F*. The curve taken on Si merely reflects the cantilever's bending, which can be used to correct the curve taken on water and to determine the correct indentation depth on the SiN membrane. The individual recorded curves are shown in **Fig. S2a**.

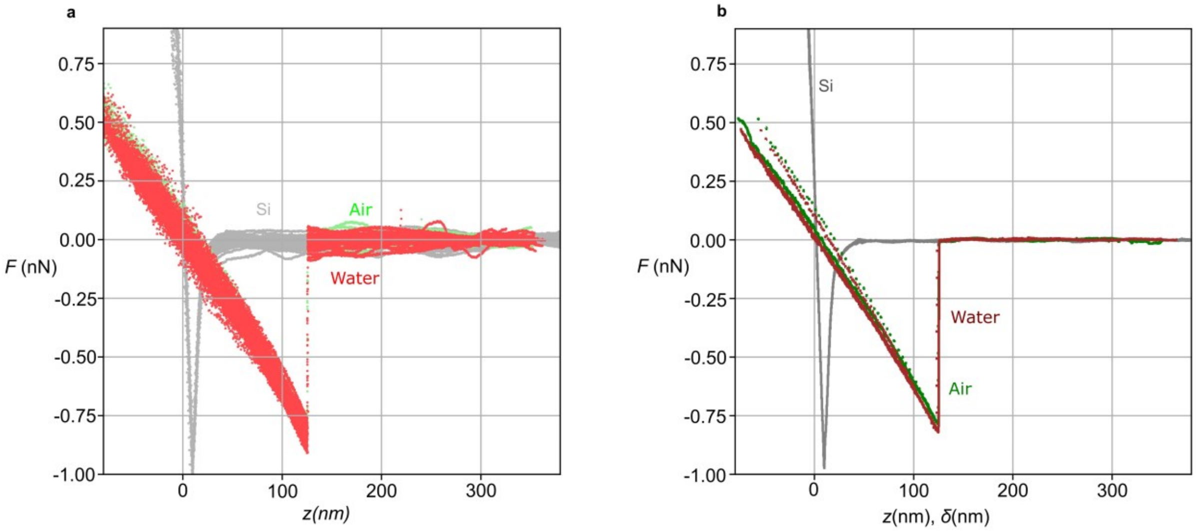

Figure S2. Contact-mode retract curves from a 10-nm thick SiN membrane deposited on a Si chip freely stretching over a square opening of 250x250 μm^2 with water adhering from below. **a** Fourty individual retract curves recorded during 1 min taken on the Si frame (gray) and on the membrane with water (20 μm away from the frame, red) and with air (20 μm away from the frame, green) underneath, showing *Z*-Piezo position *z* vs. force *F*. **b** Curves averaged from individual measurements in a without (full) and with elimination of the cantilever deflection (dotted). The height z=0 is defined at the point where the experimental curves reach $F = 0$ for the first time during the retract.

In the following, we consider the membrane as a pre-stretched, free-standing thin film and use Kirchhoff plate theory, which considers the dimensions and elastic properties of the thin membrane and establishes a relation between the applied force and resulting indentation depth based on these parameters (see **SI Section 1**). This theory has been used successfully in numerous other studies to describe the mechanics of stretched membranes.

We simplify the membrane as a circular sheet with a thickness of t_m=10 nm and a radius of R_m=125 µm. Since the ratio t_m/R_m << 1, the indentation depth δ increases approximately linearly with the applied force F [5]. We fitted the expression (see **SI Section 1**, eq. (2)) to the averaged curves shown in **Fig. S2b** after eliminating the cantilever contribution through the force-distance curves recorded on Si. This is done by assuming that the only contribution to the curves taken on Si is the cantilever deflection $d_{deflection}$ and that the bulk Si is not indented, then subtracting the curves taken on Si from the Z-piezo position curves (z) taken on the SiN membrane. This yields the tip-sample separation $\delta = z\text{-}d_{deflection}$ shown in **Fig. S2b** (dotted lines).

Assuming a Poisson ratio for SiN of 0.24 [9], we obtain a spring constant for the membrane of $k_{m,eff} = 6\cdot 10^{-3}$ Nm^{-1} and an effective elastic modulus of the SiN membrane with water adhering underneath of $E_{m,eff}$=21 TPa. This value is surprisingly large and could potentially be overinflated because we are not considering the pretension applied to the membrane, which could significantly alter its mechanical properties. In this case, eq. (2) would have to be expanded to include the pretension T [5], a quantity which would need to be determined separately:

$$F = \frac{4\pi E}{3(1-\nu^2)} \frac{{t_m}^3}{{R_m}^2} \delta + \pi T \delta$$

Predicting the shape of this deformation would necessitate precise numerical simulations, as has been done e.g. for stretched membranes in vacuum [4]. In this study, the authors show that an increase in width of the bulge caused by a tip can result from smaller in-plane tension. As can be seen in their Fig. S4, as an example, for an in-plane tension parameter of k=1, the FWHM of the bulge created by an STM tip indenting a pre-stretched membrane is around three times larger than for an in-plane tension parameter of k=100 [4].

3. Tapping phase

Phase contrasts in AFM are registered by measuring the difference between the phase angle of the excitation signal and the phase angle of the cantilever response at each position. Phase shifts are associated with the presence of inelastic tip-sample interactions, which establishes a relation between the sine of the phase shift and the power loss. The external energy E_{ext} steadily supplied to the cantilever equals the energy dissipated via hydrodynamic viscous interactions with the environment (air in our case) and via tip-sample interactions, $E_{ext} = E_{air} + E_{TS}$, where E_{TS}, is the energy dissipated through tip-sample interactions. From this, the mechanical phase φ_{mech} can be described with the following equation [10]:

$$\sin \varphi_{mech} = \frac{\omega}{\omega_0}\frac{a}{a_0} + \frac{QE_{TS}}{\pi k a a_0} \qquad (3)$$

4. Topography and mechanical phase images of a PMMA sphere submerged in water

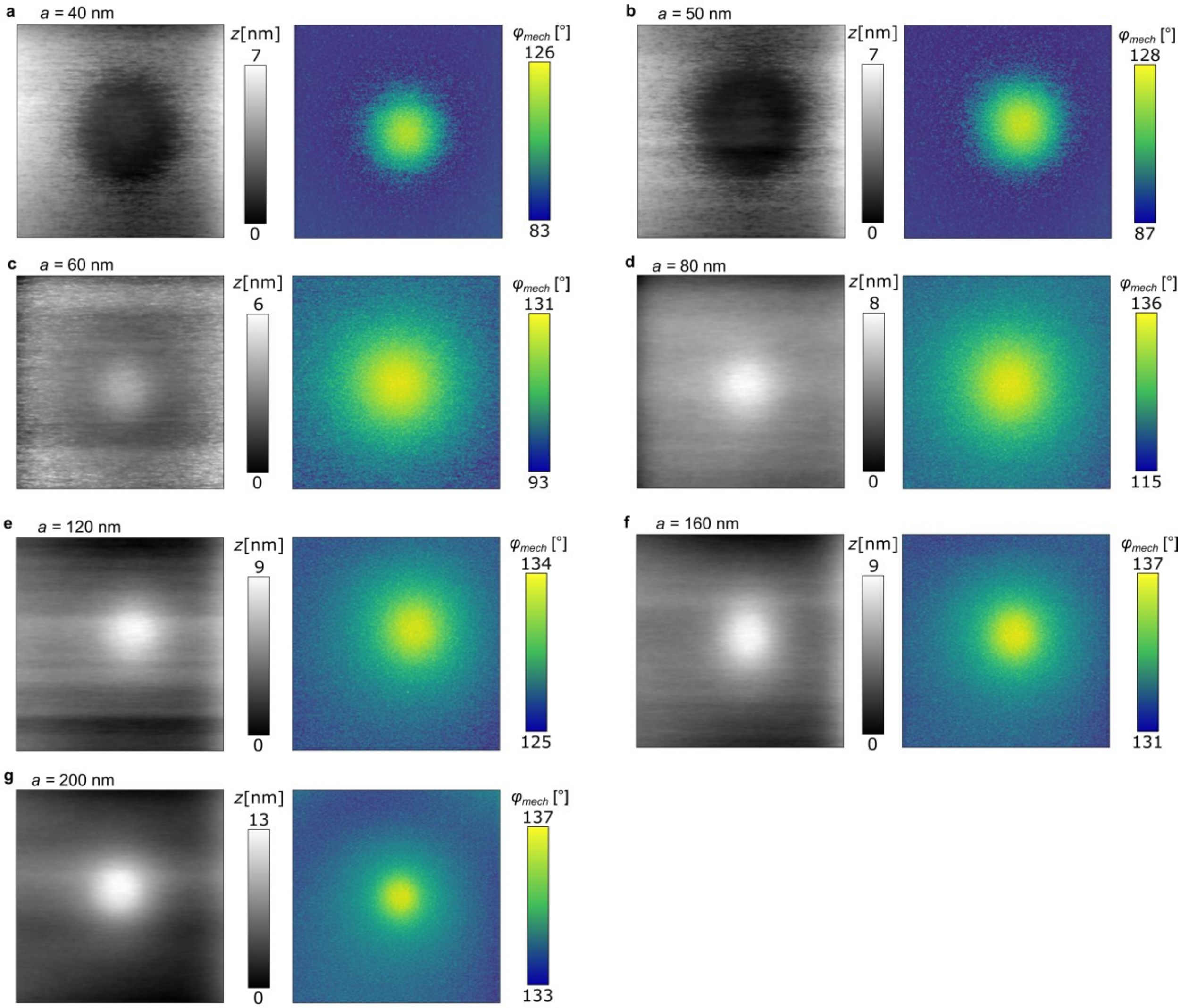

Figure S3. Measured topographies and tapping phase images of PMMA sphere underneath SiN membrane for different tapping amplitudes. **a** 40 nm. **b** 50 nm. **c** 60 nm. **d** 80 nm. **e** 120 nm. **f** 160 nm. **g** 200 nm.

5. Mechanical images of a PMMA sphere in a dried environment two days after sample preparation

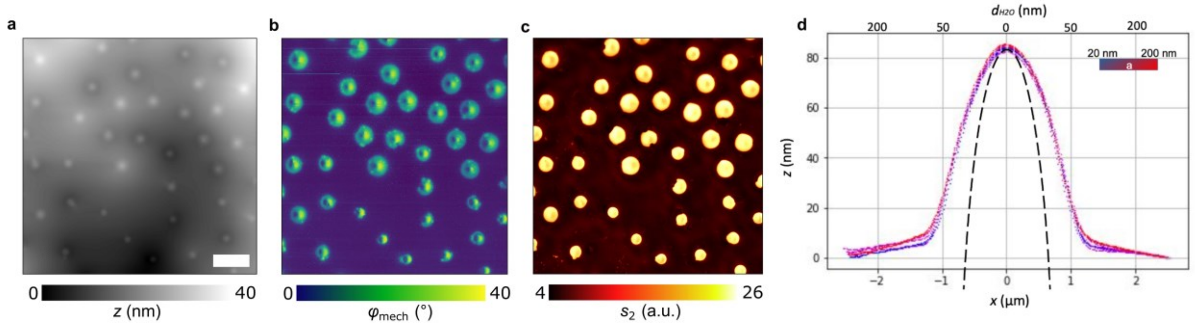

Figure S4. s-SNOM mechanical images of a dried-out SiN membrane with PMMA spheres adhering underneath a Topography, **b** tapping phase and **c** optical near-field amplitude s_2 taken on a 10-nm SiN membrane supported by a dried-out suspension of PMMA spheres supporting the membrane. The images were taken two days after sample preparation (same sample as in Fig. 3) with no water left (as has been verified through spectra not shown here). The membrane has experiences strong local deformations across the entire surface, which occurred during the drying process and were not present on the day of sample preparation. Scale bar 10 µm**. d** Topographical profiles taken along a single dried PMMA bead, showing no difference in deformation for varying tapping amplitudes and demonstrating a constant bulge of around 80 nm. We attribute this to the formation of a layer of small nanoparticles contained in the suspension that aggregate at the membrane surface during water evaporation and provide stability, preventing the membrane to be pushed downwards by the tip.

6. Extracted Profiles and Contrasts for different tapping amplitudes and amplitude setpoints

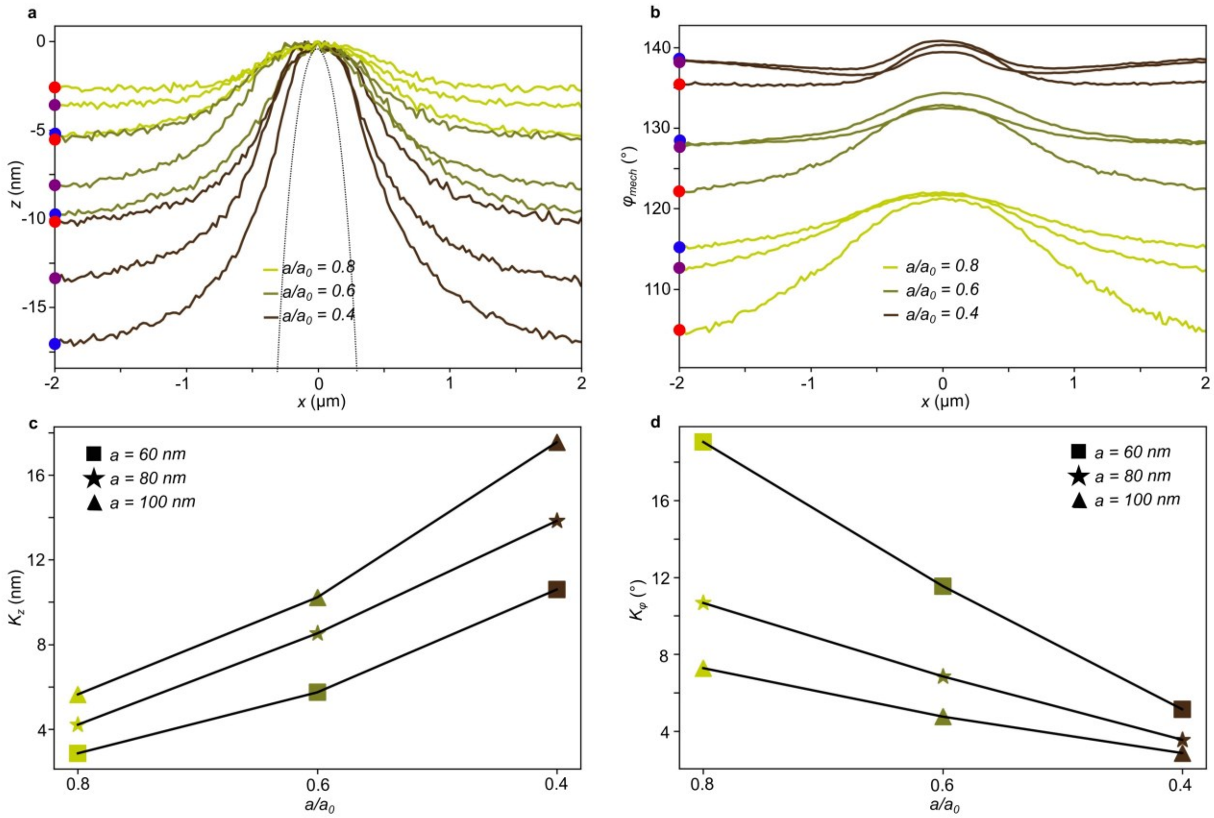

Figure S5. Extracted Profiles and Contrasts for different tapping amplitudes and amplitude setpoints a, b Extracted curves from Fig. 6 of topography and tapping phase respectively measured with different amplitude setpoints (yellow for $a/a_0 = 0.4$, dark yellow for $a/a_0 = 0.6$, brown for $a/a_0 = 0.8$), each measured with three different tapping amplitudes a of 60 nm, 80 nm and 100 nm. Scale bar 1 μm. **c, d** Contrast between measured topography and tapping phase far away (2 μm) from the sphere and on top of the sphere as a function of amplitude setpoint for different tapping amplitudes a (square shapes for a = 60 nm, star shapes for a = 80 nm, triangle shapes for a = 100 nm). All data was acquired using a probe with radius r = 60 nm.

7. Single-wavelength imaging of PMMA spheres in water

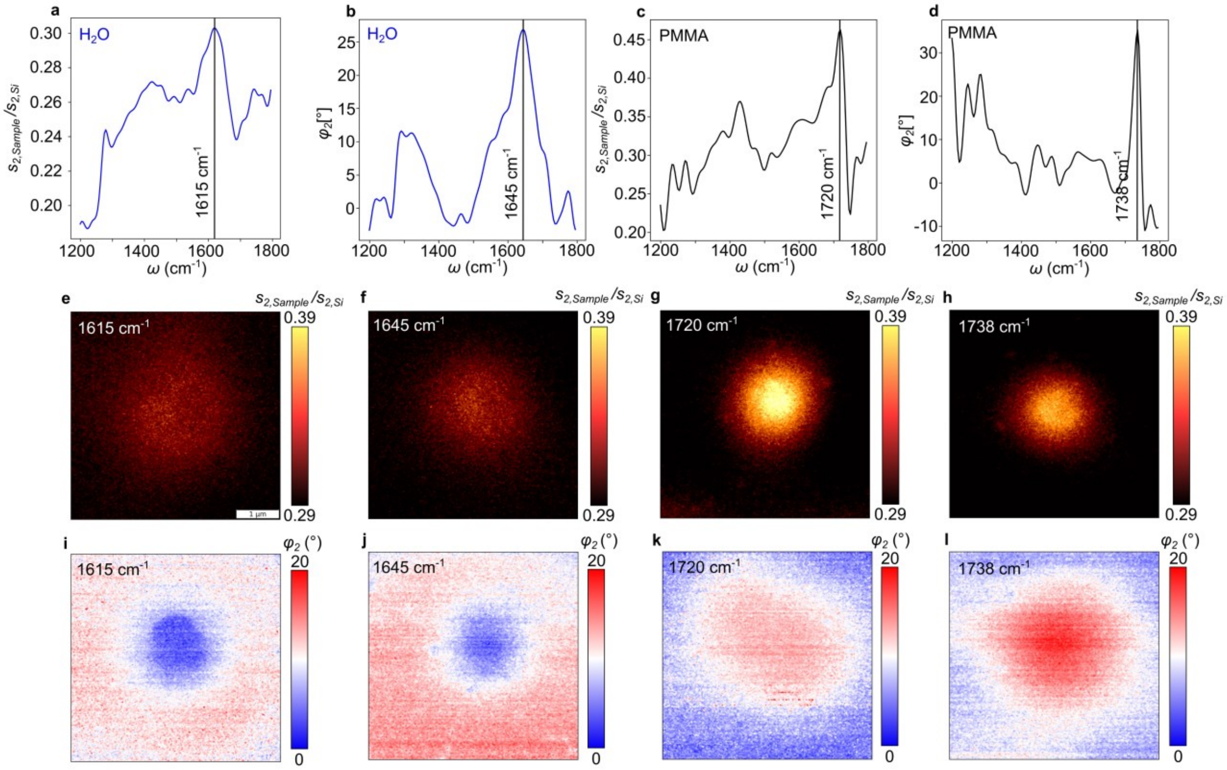

Figure S6. Spectra and nano-imaging of a PMMA sphere in water a, b Nano-FTIR s_2 amplitude and φ_2 phase spectra of water underneath a 10 nm SiN membrane. **c, d** Nano-FTIR s_2 amplitude and φ_2 phase spectra of a PMMA sphere underneath a 10 nm SiN membrane taken at the center of the sphere to ensure that no water layer is present. **e-l** Single-wavelength images s_2 amplitude and φ_2 phase taken at four-indicated wavelengths, illustrating how contrasts vary vs frequency according to the spectra shown in a-d.

Figure S6. Spectra and nano-imaging of aPMMA sphere in water a, b Nano-FTIR s2 amplitude and phi2 phase spectra of water underneath a 10 nm SiN membrane. **c, d** Nano-FTIRs2 amplitude and phi2 phase spectra of a PMMA sphere underneath a 10 nm SiN membrane, taken at the sphere's center to ensure that no water layer is present. **e-l** Single-wavelength images of optical s2 amplitude and phi2 phase taken at four wavelengths as indicated , illustrating how contrasts vary vs frequency according to the spectra shown in a-d.

8. Imaging of aggregated amyloid-beta protein strands in water

A frozen suspension containing amyloid-beta peptides was thawed at 0°C for 1/2 h, then 1:20 diluted with water before being pipetted it into the liquid cell (see **Experimental section**), then sealed after a deposition time of 45 min. s-SNOM images taken in 12 min sequence exhibit a rather compact 1x0.5 μm^2 protein aggregate that was stable in time for hours. Interesting is the high spatial resolution attained, of <30 nm as can be estimated from repeated detail in grainy structures around the object (pixel size about 15 nm). The body of the aggregated object exhibits a rather flat topography and likewise, quite homogeneous contrasts in both infrared and φ_{mech} images, as compared to the surrounding.

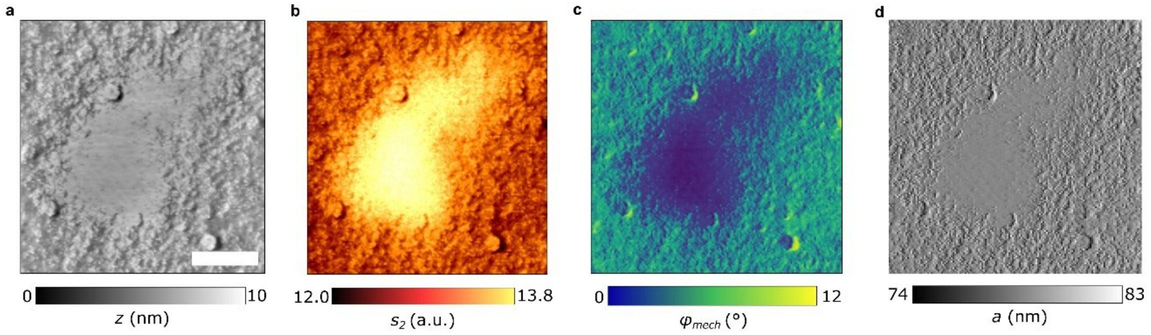

Figure S7. s-SNOM images of amyloid-beta protein aggregates in water a Topography, **b** s_2 infrared amplitude ("white light" spectral average), **c** tapping phase and **d** tapping amplitude (error image) of aggregated amyloid-beta protein strands adhering in water to a 10-nm SiN membrane, with initial tapping amplitude set to a = 80 nm. Scale bar 500 nm.

References

[1] J. Tamayo and R. García, "Deformation, Contact Time, and Phase Contrast in Tapping Mode Scanning Force Microscopy," *Langmuir,* vol. 12, no. 18, p. 4430–4435, 1996.

[2] A. Khan, J. Philip and P. Hess, "Young's modulus of silicon nitride used in scanning force microscope cantilevers," *Journal of Applied Physics,* vol. 95, no. 4, p. 1667–1672, 2004.

[3] A. A. Love, "The Small Free Vibrations and Deformation of a Thin Elastic Shell," *Journal of Microelectromechanical Systems,* vol. 179, no. , 1888.

[4] K. Elibol, S. Hummel, B. C. Bayer and J. C. Meyer, "New imaging modes for analyzing suspended ultra-thin membranes by double-tip scanning probe microscopy," *Scientific reports,* vol. 10, no. 1, p. 4839, 2020.

[5] A. Castellanos-Gomez, M. Poot, G. A. Steele, H. S. J. van der Zant, N. Agraït and G. Rubio-Bollinger, "Elastic properties of freely suspended MoS2 nanosheets," *Advanced materials (Deerfield Beach, Fla.),* vol. 24, no. 6, p. 772–775, 2012.

[6] J. Tao, W. Shen, S. Wu, L. Liu, Z. Feng, C. Wang, C. Hu, P. Yao, H. Zhang, W. Pang, X. Duan, J. Liu, C. Zhou and D. Zhang, "Mechanical and Electrical Anisotropy of Few-Layer Black Phosphorus," *ACS nano,* vol. 9, no. 11, p. 11362–11370, 2015.

[7] C. Di Giorgio, E. Blundo, G. Pettinari, M. Felici, F. Bobba and A. Polimeni, "Mechanical, Elastic, and Adhesive Properties of Two-Dimensional Materials: From Straining Techniques to State-of-the-Art Local Probe Measurements," *Advanced Materials Interfaces,* vol. 9, no. 13, 2022.

[8] A. Janshoff and C. Steinem, "Mechanics of lipid bilayers: What do we learn from pore-spanning membranes?," *Biochim Biophys Acta.,* vol. 1853, no. 11, 2015.

[9] B. A. Walmsley, Y. Liu, X. Z. Hu, M. B. Bush, J. M. Dell and L. Faraone, "Poisson's Ratio of Low-Temperature PECVD Silicon Nitride Thin Films," *Journal of Microelectromechanical Systems,* vol. 16, no. 3, 2007.

[10] J. Tamayo and R. García, "Relationship between phase shift and energy dissipation in tapping-mode scanning force microscopy," *Applied Physics Letters,* vol. 73, no. 20, p. 2926–2928, 1998.
